# Critical incidents and post-traumatic stress symptoms among experienced registered nurses during the COVID-19 pandemic: A cross-sectional study

**DOI:** 10.1016/j.ijnsa.2024.100194

**Published:** 2024-03-27

**Authors:** Sara Melander, Oili Dahl, Ann-Charlotte Falk, Veronica Lindström, Erik Andersson, Petter Gustavsson, Ann Rudman

**Affiliations:** a School of Health and Welfare, Dalarna University, Falun, Sweden; b Division of Psychology, Department of Clinical Neuroscience, Karolinska Institutet, Stockholm, Sweden; c Division of Nursing, Department of Neurobiology, Care Sciences and Society, Karolinska Institutet, Stockholm, Sweden; d Department for Health Promotion Science, Sophiahemmet University, Stockholm, Sweden; e Department of Nursing, Umeå University, Umeå, Sweden; f Department of Ambulance service, Region Västerbotten, Umeå, Sweden

**Keywords:** COVID-19, Nurses, Occupational health, Post-traumatic stress disorders, Critical incidents, Work environment, Psychological, Mental Health, Quantitative methodology

## Abstract

**Background:**

Registered nurses working on the frontline during the COVID-19 pandemic encountered significant challenges, including exposure to critical incidents. Critical incidents refer to sudden unexpected clinical events that surpass an individual's ability to cope, leading to considerable psychological distress, which could potentially result in the development of post-traumatic stress disorder symptoms. Research has shown a high prevalence of post-traumatic stress disorder symptoms among healthcare workers, particularly those in close contact with COVID-19 patients.

**Objective:**

To assess the levels of post-traumatic stress symptoms among registered nurses in relation to exposure to working conditions during the COVID-19 pandemic, such as how much their work was affected by the pandemic, re-deployment, working hours hindering sufficient recovery between shifts and critical incidents.

**Design:**

Cross sectional study.

**Setting(s):**

The registered nurses working in multiple health care services covering all 21 geographic regions in Sweden.

**Participants:**

A total of 1,923 registered nurses, who are part of a Swedish national cohort and have been followed since their nursing education, were invited to participate in a survey in late September 2021 (15 to 19 years post graduation).

**Methods:**

The data were analyzed using descriptive statistics, unpaired *t*-tests, and one-way analysis of variance. Cohen's d was employed to quantify differences in mean levels between subgroups.

**Results:**

The response rate were 56.5 %. Over 50 % of experienced registered nurses reported significant disruptions to their work environments. In total, 85 % of registered nurses were exposed to at least one critical incident in their work during the pandemic, with 60 % facing organisational changes and nearly 50 % experiencing emotionally distressing situations. The exposure to work situations involving critical incidents consistently demonstrated strong associations with higher levels of post-traumatic stress disorder symptoms compared to those not exposed, with effect sizes ranging from moderate to high.

**Conclusions:**

This study underscores the profound impact that working conditions, such as redeployment and exposure to critical incidents, have on the mental health of registered nurses. We offer valuable insights into registered nurses’ pandemic-related challenges, highlighting the need for support and interventions to prevent and manage critical incidents, ultimately promoting their well-being. We also highlight the significance of thorough workforce readiness planning for future pandemics and other challenging health care scenarios, such as staff shortage.

## What is already known about the topic

• Health care services, particularly those in emergency and intensive care settings where critical incidents are more common, face a risk of events that can potentially impact the well-being of personnel.
• Nurses, such as frontline health care providers, have faced substantial consequences affecting both their working conditions and their health, including symptoms of post-traumatic stress disorder.
• In response to the pressing demand for hospital beds, personnel were frequently reassigned from various departments and rapidly adapted to unfamiliar work environments.

## What this paper adds

• Registered nurses exposed to non-voluntary redeployment reported higher levels of post-traumatic stress disorder symptoms compared to their non-exposed counterparts.
• A significant relation was found between insufficient recovery between shifts and levels of post-traumatic stress disorder symptoms.
• In this study, 85 % of experienced registered nurses encountered at least one critical incident, with an average of 4.5 critical incidents (distressing clinical event) per nurse.

## 1 Introduction

The COVID-19 pandemic caused a burden on the health care system and disrupted ordinary health care (Arsenault et al., 2022). On the frontline of health care services during the pandemic, nurses were the largest group (59 %) of health care providers, and they are essential to the delivery of health care and prevention of illness (WHO, 2020). Registered nurses’ (RNs’) working conditions during the pandemic caused increased illness, such as post-traumatic stress symptoms (Carmassi et al., 2020; Galanis et al., 2021; Johnson et al., 2020; Stafseth et al., 2022). In developing interventions to prevent health issues and increased intention to leave among RNs, there is a need for more knowledge about critical incidents (distressing clinical events) and other working conditions that nurses were exposed to during the pandemic throughout the health care system, as well as the effects these critical incidents had on RNs’ health. Therefore, the overall aim of this study was to assess the levels of post-traumatic stress symptoms among RNs in relation to exposure to working conditions during the COVID-19 pandemic by examining how much their work was affected by the pandemic, re-deployment, working hours hindering sufficient recovery between shifts, and critical incidents.

### 1.1 Background

During the COVID-19 pandemic, the health care system faced overwhelming challenges. RNs, alongside other health care professionals, were on the frontline, which significantly impacted their working environment and affected their workload (Galanis et al., 2021) and health (Gómez-Ochoa et al., 2021). To address the urgent need for hospital beds, health care personnel were often transferred from different wards and quickly introduced to work in the intensive care unit (Ahlsson, 2020). Critical incidents refer to abrupt or unforeseen clinical events that surpass an individual's ability to cope, leading to considerable psychological distress and are defined as “a sudden unexpected event that has an emotional impact sufficient to overwhelm the usually effective coping skills of an individual and cause significant psychological stress” (Caine and Ter-Bagdasarian, 2003, p. 60). Exposure to critical incidents (such as the current response to COVID-19) increases the already heavy workload of physicians and nurses and can potentially lead to associated trauma (Caldas et al., 2021). Diverse working conditions during the pandemic, such as increased workloads, staff shortages, redeployments, a lack of adequate personal protective equipment, and the emotional toll of continuously witnessing patient suffering and death placed significant strain on RNs (Romero-García et al., 2022).

It is known that health care personnel working in health care services such as emergency and intensive care with a high risk of unexpected patient events have an increased risk of experiencing adverse incidents (Wu et al., 2020). This increase in workload and reorganisation heightens the risk of exposure to critical incidents for the personnel working in health care. When health care providers become involved in unanticipated patient events, medical errors, or patient-related injuries, they subsequently experience trauma as a result (Scott et al., 2009). Often, these individuals feel a personal responsibility for the patient's outcome, grappling with feelings of failure and self-doubt regarding their clinical skills and knowledge base. Typical incidents can include conflicts, abuse, unanticipated death, and questionable practices (Michael and Jenkins, 2001). Critical incidents that registered nurses have encountered or witnessed in the workplace can impact their health or well-being, work ability, and the effectiveness of the health care organization (Karanikola et al., 2022). A systematic review conducted by Buhlmann et al. (2021) emphasised the significant psychological impact that critical incidents have on health care personnel, potentially leading to profound emotional distress. Encountering critical incidents can trigger adverse emotional responses, leaving RNs deeply distressed, with feelings of helplessness, intense sorrow, and a diminished self-worth. Such experiences can also manifest themselves as physical symptoms including nausea, elevated body temperature, rapid heart rate, perspiration, and tremors, along with extended periods of hyper-vigilance and recurrent flashbacks stemming from the critical incident (Buhlmann et al., 2021). As a consequence, RNs may exhibit typical signs of post-traumatic stress disorder. According to The Diagnostic and Statistical Manual of Mental Disorders, Fifth Edition (DSM-5), the central criteria for post-traumatic stress disorder include exposure to an extraordinary stressor beyond the realm of typical stressors, persistent re-experiencing of the events through intrusive memories and flashbacks, and consistent efforts to avoid trauma-related stimuli (Friedman et al., 2021. p. 19).

Research conducted during the COVID-19 outbreak has shown that approximately one-third of health care workers and public service providers experienced post-traumatic stress disorder symptoms, with an additional 20 % reporting anxiety and depression (Johnson et al., 2020). Furthermore, health care workers who worked in high-risk wards, such as intensive care units, or directly with COVID-19 patients, reported significantly higher levels of post-traumatic stress disorder symptoms and burnout compared to those who did not (Carmassi et al., 2020; Galanis et al., 2021; Johnson et al., 2020; Stafseth et al., 2022). However, there is a lack of research that specifically examines critical incidents and post-traumatic symptoms amo ng nurses working in different parts of the health care system. Therefore, gaining a deeper understanding of RNs' exposure and reactions could contribute to the development of interventions aimed at preventing further health care issues and reducing the intention to leave among nurses.

### 1.2 Objective

To assess the levels of post-traumatic stress symptoms among RNs in relation to exposure to working conditions during the COVID-19 pandemic, we examined how much their work was affected by the pandemic, re-deployment, working hours hindering sufficient recovery between shifts, and critical incidents.

### 1.3 Research questions

1. What were the levels of post-traumatic stress symptoms among RNs during the late stages of the COVID-19 pandemic?
2. To what extent were the RNs exposed to working conditions during the COVID-19 pandemic, and how strongly was this exposure associated with levels of post-traumatic stress disorder symptoms? Specifically, were higher levels of post-traumatic stress disorder symptoms more prevalent among those who, during the COVID-19 pandemic, were exposed to the following:
  1. Work affected by the pandemic,
  2. Re-deployment,
  3. Working hours hindering sufficient recovery between shifts, and
  4. Critical incidents?

## 2 Methods

### 2.1 Study design and participants

The study design was cross-sectional. A Swedish national cohort of RNs were invited to answer a survey that was send out to them by letter in the last week of September 2021 (15 to 19 years post graduation). The majority answered the questionnaire within the first few weeks. Two reminders were sent out in October and November, and the data collection was finally closed the first week of January 2022. The data were collected by surveys administered by Statistics Sweden and could be answered in paper form sent back in a prepaid envelope or online. The survey consisted of 98 questions including 22 open-ended questions. Two reminders were sent out, and the survey could be answered in paper form or online. The RNs were part of the LANE (Longitudinal Analysis of Nursing Education/Entry in Work-Life) study (Rudman et al., 2010). When the cohorts were formed, the participants were in their nursing programme, and those who joined the cohorts did not deviate from the student population to any significant extent. For details, see Rudman et al. (2010).

### 2.2 Measurement

All data were self-reported. The variables used to measure post-traumatic stress disorder symptoms and critical incidents in the analyses were derived from established instruments, as described below. The assessments of RNs’ novel and diverse work situations during the COVID-19 pandemic were conducted using study-specific questions developed by the research group. These questions underwent pilot testing among RNs working in different areas of the health care services. The pilot test confirmed that questions surrounding critical incidents were acceptable and that the list of situations that nurses may have been exposed to during the COVID-19 pandemic was relevant. In this study, items related to COVID-19 work (i.e., how much their work was affected by the pandemic, re-deployment, and working hours hindering sufficient recovery between shifts) were collected for 3-month retrospective periods, and the detailed questions used are presented in Supplementary Material Table S1. Since the trajectory of the pandemic was largely unknown, we believed that three-month periods would provide sufficient detail without imposing too much burden on respondents. In addition, the pilot test confirmed that dividing the questions related to COVID-19 work (see Supplementary Material Table S1) into 3-month periods was feasible. In our pretesting of the instrument, nurses were able to recall relevant events and report their experiences.

### 2.3 The posttraumatic stress disorder checklist

In this study, the RNs were asked to assess their levels of post-traumatic stress disorder symptoms over the past month. Reactions to specific self-defined work-related incidents were measured using a validated instrument PCL-5 (Post-Traumatic Stress Disorder Checklist for DSM-5) (Arnberg et al., 2014; Blevins et al., 2015). The PCL-5 includes 20 self-assessment items that directly correspond to the diagnostic and statistical manual for post-traumatic stress disorder symptoms. The participants in this study answered in a translated version of the instrument into Swedish. In this study, the RNs were asked to think about the most stressful events at work throughout the pandemic and how much this affected them during the last month. The instructions read as follows:

“The following questions are about stressful events in your work during the pandemic.

*A stressful event often involves death or risk of death, serious injury or sexual violence. It can be something that happened to you personally, something you witnessed or something you found out happened to a close relative or colleague.*

*Think about the most stressful events experienced during the pandemic that are affecting you right now. Then read each of the sub-questions and tick how much you were bothered during the last month.”*

They were instructed to rate these statements/claims on a five-point scale with the response alternatives “Extreme”, “Pretty Much”, “Moderate”, “Little”, or “Not at all”. Item responses were coded from “Not at all” (0) to “Extreme” (4) and summated to acquire a total post-traumatic stress disorder score ranging between 0 and 80. Items were sorted into four subscales, henceforth called symptom clusters; i.e., intrusive memories (items 1–5), avoidance behavior (items 6–7), changes in negative emotions and mood (items 8–14), and physiological activation (items 15–20). Scale scores have been computed as mean scores (0–4) to compare levels among these subscales. In addition, as the instructions to the respondents were adapted to a survey format focusing on traumatic events during the COVID-19 pandemic, some basic psychometric analyses of the present data were performed. First, the internal structure was tested using standard procedures for confirmatory factor analysis (Brown, 2015). Two models were tested (using standard procedures in Mplus 8.5 for ordinal item data): one with four factors reflecting the four subscales and another reflecting the suggested use of the total PCL-5 score. Both models showed adequate fit to the data according to multiple fit indices and their suggested cut-offs (the four-factor model: χ²(df164) =1241, *p* < .001, CFI (Comparative Fit Index) = 0.976, RMSEA (Root Mean Square Error of Approximation) = 0.059, SRMR (Standardized Root Mean Square Residual) = 0.035; and one-factor model: χ²(df170) =2465, *p* < .001, CFI = 0.949, RMSEA = 0.085, SRMR = 0.056). Second, internal consistency reliability was computed using Cronbach's alpha, estimated at 0.90 for the total scale (and varied between 0.84 to 0.90 for the four subscales). A cut-off of 31 points has been recommended (Weathers et al., 2013) to reflect a possible diagnosis of post-traumatic stress disorder; hence, this was used to estimate its prevalence.

### 2.4 Critical incidents

The instrument measuring the prevalence of critical incidents was based on earlier research concerning critical incidents and definitions of traumatic events generated from a population working in healthcare settings, and the items were based on work done by O´Conner (2003). The instrument used in this study contains items measuring occurrences of traumatic events in health care. The respondent had the opportunity to rate if they had been exposed to these events during the COVID-19 pandemic with the response scale, “Yes, many times”, “Yes, a few times”, “Yes, one time” and, “No”. In the statistical analyses, the response scale was categorised to “Yes (one time, a few times and many times)” or “No”.

### 2.5 Statistical methods

The data analyses used in this study were descriptive statistics, an unpaired *t*-test, and a one-way analysis of variance (ANOVA). The total PCL-5 score was used as the primary outcome variable in all analyses, while the analyses involving the PCL-5 subscales were exclusively conducted for supplementary purposes. Differences in mean levels between groups were transformed to effect sizes, Cohen's *d.* The effect sizes according to Cohen (1998) were categorised as small effect size =0.2, medium effect size= 0.5 and large effect size = 0.8. The significance level was set at 0.05, and internal dropout was 3.6 % for post-traumatic stress disorder symptoms, 4.9 % for work affected by COVID-19, 11.4 % for re-deployment, 5.1 % for working hours hindering sufficient recovery between shifts and 2.3 % for critical incidents. No imputation was made on internal dropout. The analyses were completed in IBM SPSS version 28 (SPSS Inc, 2019).

### 2.6 Ethical approval

Ethical approval has been given by the Swedish ethical review authority (Dnr 01–045, 2006/973–32, 2021–00,958, 2022–04,898–02). To guarantee confidentiality, the data collection has been administered by Statistics Sweden. The de-identified research material is kept confidential at the Department of Clinical Neuroscience, Karolinska Institutet, Stockholm, Sweden. All participants gave informed consent, with assurance that they could withdraw from the study at any time.

## 3 Results

### 3.1 Basic characteristics of the sample

For the data collection in 2021, 3958 (91.7 %) participants were eligible, and out of these 2237 (56.5%) responded to the survey. In Supplementary Material Figure S1, a flow chart of recruitment and participation in the study is shown. The sample used in the analyses consisted of the RNs who answered that they worked in health care during the COVID-19 pandemic and answered questions regarding their work life. In terms of both availability and response rates, no statistically significant difference was found among men (90.0 %) or women (91.9 %) or with respect to previously high (91.6 %) or low (92.2 %) levels of self-rated health. However, between age groups, the response frequency was statistically significant, with older participants having responded to a somewhat higher extent.

The RNs worked in all 21 geographic regions in Sweden. A majority (95.6 %, *n* = 1921) did not change regions during the pandemic, while 4 % voluntarily worked in other regions. Another 0.3 % were ordered by their employer to change region. The demographic characteristics of the participants and comparisons of mean levels of posttraumatic stress disorder are shown in Supplementary Material Table S2. There was no significant difference in mean levels of posttraumatic stress disorder symptoms regarding gender, age, specialist education, current position, employer, or types of employment. In terms of workplace context, RNs employed in reception and primary health care services had a significantly lower mean levels of symptoms related to posttraumatic stress disorder compared to RNs working in nursing wards, nursing homes, operating theatre/intensive care, research/education, and other workplace settings.

### 3.2 Current posttraumatic stress disorder symptoms

In Table 1 the mean level for the posttraumatic stress disorder total scale and subscales are presented and 3.9 % of the RNs had levels above the cut-off. Among the four subscales of posttraumatic stress disorder, the highest mean was found for the “Physiological activation” subscale, and the lowest mean was for the “Avoidance” subscale.

**Table 1:** Mean of post-traumatic stress disorder symptoms divided into symptom clusters. Subscales (clusters) range between 0 and 4 and total scale range between 0 and 80. Number of participants ranges between 1853 and 1868.

Post-traumatic stress disorder symptoms (PCL-5*)		Mean	SD
Subscale (clusters)**			
	Intrusive memories	0.264	0.517
	Avoidance behavior	0.237	0.607
	Negative emotions and mood	0.273	0.539
	Physiological activation	0.338	0.565
Total scale	PCL-5	5.693	9.783
		*n*	*%*
Total scale cut-off	PCL-5 (*>*30)	72	3.9

*PCL-5 (The Post-Traumatic Stress Disorder Checklist for DSM-5).

**To compare levels among these subscales, scale scores have been computed as mean scores (0–4). *n*= number of participants.

### 3.3 *Work affected by the* COVID-19 *pandemic*

**Fig. 1:**
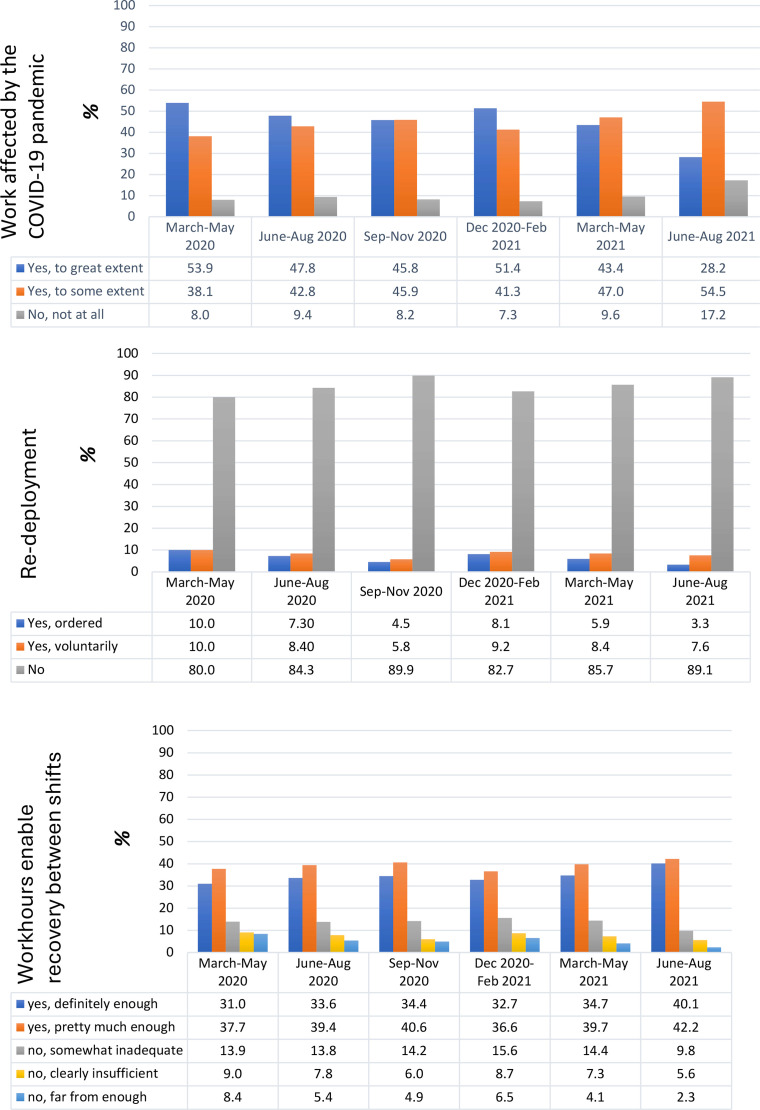
Registered nurses’ retrospective ratings of different working conditions during different phases of the COVID-19 pandemic. Data presented as proportions of RNs with different degrees of exposure. Number of participants range between 1703 and 1888 related to internal drop-out.

During the time period between March 2020 and August 2021, approximately 20–50 % of the RNs reported that work was greatly affected by the pandemic (Fig. 1). The RNs who reported that their work had been greatly affected by the pandemic later reported significantly higher levels of posttraumatic stress disorder symptoms (Table 2). This association was shown for all six time periods of the pandemic.

**Table 2:** Relationship between post-traumatic stress disorder symptoms (PCL-5* Total Score) and the three exposure variables: a) how much work was affected, b) re-deployment, c) insufficient recovery between shifts, across six phases of the COVID-19 Pandemic. For group sizes see Fig. 1.

	March-May 2020		June-Aug 2020		Sep-Nov 2020		Dec 2020-Feb 2021		March-May 2021		June-Aug 2021	
	*M*	SD	*M*	SD	*M*	SD	*M*	SD	*M*	SD	*M*	SD
a) How much their work was affected												
Yes, great extent	7.17	10.70	7.37	10.90	7.24	10.63	7.33	10.80	7.60	10.90	7.72	11.50
Yes, some extent	3.83	8.00	3.91	7.86	4.23	8.40	3.84	7.90	4.17	8.40	4.95	8.80
No, not at all	4.50	8.70	4.54	8.95	4.62	9.50	3.87	8.18	3.88	7.90	4.26	8.35
*F*	25.884		28.454		21.544		30.62		29.808		17.804	
*p*	*<*0.001		*<*0.001		*<*0.001		*<*0.001		*<*0.001		*<*0.001	
b) Re-deployment												
Yes. ordered	9.30	12.39	9.00	11.88	9.80	11.62	8.83	12.11	10.62	13.64	10.8	14.83
Yes. voluntarily	6.30	9.91	7.14	11.72	7.27	11.16	6.82	10.40	5.00	8.30	6.44	10.50
No	5.00	9.00	5.00	9.00	5.28	9.30	5.21	9.30	5.36	9.50	5.35	9.40
*F*	15.969		12.331		9.547		10.213		14.202		8.873	
*p*	*<*0.001		*<*0.001		*<*0.001		*<*0.001		*<*0.001		*<*0.001	
a) Hindrance of sufficient recovery between shifts												
Yes, definitely enough	2.00	4.90	2.21	5.25	2.14	5.00	2.08	5.31	2.19	5.41	2.65	5.80
Yes, pretty much enough	4.80	8.40	5.11	8.65	5.43	9.38	4.40	7.56	4.90	8.00	6.03	9.37
No, somewhat inadequate	7.91	10.64	8.84	11.28	9.39	10.76	8.50	10.77	9.46	11.39	9.20	11.00
No, clearly insufficient	10.83	12.12	11.6	12.80	11.52	12.17	12.71	13.37	13.07	13.29	12.91	13.34
No, far from enough	13.45	15.13	13.24	14.83	13.86	15.57	13.96	14.52	14.79	15.75	16.13	18.97
*F*	70.358		63.479		64.187		86.997		81.715		58.063	
*p*	*<*0.001		*<*0.001		*<*0.001		*<*0.001		*<*0.001		*<*0.001	

* The Post-Traumatic Stress Disorder Checklist for DSM-5. *M*= mean, SD= Standard Deviation.

### 3.4 Re-deployment

During the first three months of the pandemic (March to May 2020), 20 % of the RNs were re-deployed (Fig. 1). These levels fluctuated somewhat during different phases of the pandemic, but in general they decreased over time. Re-deployed RNs (who were ordered to move) later reported significantly higher levels of posttraumatic stress disorder symptoms (Table 2) across all time periods.

### 3.5 Working hours hindering sufficient recovery between shifts

Between March-May 2020, RNs reported the highest prevalence of exposure to demanding working hours that hindered sufficient recovery between shifts. During this early phase of the pandemic, over 17 % revealed they were exposed to working hours that hindered sufficient recovery between shifts (Fig. 1). The second highest prevalence of exposure was found during Dec 2020-Feb 2021. Over all time periods, there was a significant relation between working hours that enabled enough recovery and levels of posttraumatic stress disorder symptoms (Table 2).

### 3.6 Critical incidents

The prevalence of RNs’ exposure to different critical incidents during the COVID-19 pandemic ranged between 12 % and 60 % (Table 3). Out of a total of 1894 RN, 85 % were exposed to at least one critical incident during the pandemic. On average, RNs were exposed to 4.5 critical incidents (SD 3592; range 0 to 14) during the pandemic. For all incidents, there were significant differences in levels of posttraumatic stress disorder symptoms. The effect size (Cohen´s *d*) for these differences were mostly medium to large (ranging between 0.35–0.80). The incidents that had the strongest associations with posttraumatic stress disorder symptom levels; i.e., the largest effect sizes, were events where a patient reminded RNs of themselves, a family member, or a friend. In other cases, similar effects occurred when RNs felt unsure of their responsibilities in an emergency care situation or received incorrect or insufficient information, hindering their ability to provide adequate care in an emergency situation.

**Table 3:** Prevalence of exposure to critical incidents and differences in the levels of post-traumatic stress symptoms. Number of participants range between 1878 and 1890, *M*=mean, SD=Standard Deviation. .

	PTSD* symptoms Exposure to critical incidents	Prevalence of incidents**	Levels of PTSD symptoms				Test of mean differences		
		*%*	*M*	SD	*M*	SD	*t*	*p*	Cohen's *d*
1	Seen or cared for someone you know for severe injury or disease	21.7	9.6	12.0	4.6	8.8	7.7	*<*0.001	0.513
2	Seen or cared for a child that was severely injured or sick	19.6	8.5	12.0	5.0	9.0	5.2	*<*0.001	0.356
3	Seen or cared for a patient that has tried to take their own life or consciously hurt themself	32.4	8.0	11.2	4.7	8.8	6.7	*<*0.001	0.360
4	Have experienced or seen violence, threats of violence, or other aggression from patients or relatives	40.2	8.3	11.4	3.9	8.0	9.3	*<*0.001	0.467
5	A patient's traumatic or tragic death	40.3	9.1	12.0	3.4	7.1	11.8	*<*0.001	0.611
6	Difficult or unsuccessful resuscitation	21.9	9.2	12.6	4.7	8.6	6.9	*<*0.001	0.476
7	A traumatic or tragic event in which a patient reminded you of yourself, a family member, or a friend	26.8	10.7	12.5	3.8	7.8	11.6	*<*0.001	0.742
8	Events that involved very emotionally upset or grieving relatives	47.5	8.8	11.7	2.9	6.4	13.4	*<*0.001	0.638
9	Feeling helpless because nothing could be done to help a patient	49.1	8.5	11.5	2.9	6.5	13.0	*<*0.001	0.607
10	Feel unsure of what responsibilities or powers you had in an emergency care situation	25.4	10.9	13.1	3.9	7.6	11.0	*<*0.001	0.746
11	Received incorrect or insufficient information to be able to provide adequate care in an emergency situation	25.1	11.0	13.2	3.9	7.5	11.2	*<*0.001	0.774
12	Did not get immediate access to doctors or help from colleagues when you needed it	35.5	9.7	12.5	3.4	6.9	12.1	*<*0.001	0.638
13	Organisational change that involved changed working conditions	59.9	7.5	11.0	3.0	6.7	10.9	*<*0.001	0.471
14	Other traumatic events during COVID-19, Open-ended responses	12.4	11.3	12.9	4.1	8.3	6.6	*<*0.001	0.803

*PTSD, post-traumatic stress disorder.

**Critical incidents, YES, have experienced the incident one or more times.

### 3.7 Secondary analysis

The dose-response association between critical incidents and levels of post-traumatic stress disorder symptoms was also examined using all four levels of exposure to each critical incident and Post-Traumatic Stress Disorder Checklist DSM-5 total scale scores. Analysis of variances (ANOVA) was computed, and the corresponding results are shown in Supplementary Material Table S3. In general, more exposure was reflected in higher posttraumatic stress disorder symptom levels. Effect sizes reflecting differences between no exposure and being exposed several times ranged between 0.567 and 1.940 (see Supplementary Material Table S3).

The dose-response analyses on the influence of exposure to critical incidents and levels of post-traumatic stress symptoms were also performed on each of the four subscales, respectively. Results for the four subscales are presented in the Supplementary Material Table S4-S7. Associations between exposure to critical incidents and the four post-traumatic stress disorder symptom subscales were generally found to be less pronounced for the physiological activation subscale (i.e., yielding lower effect sizes).

## 4 Discussion

The aim of this study was to examine the levels of posttraumatic stress disorder symptoms among RNs in relation to exposure to occupational-related factors, including how much their work was affected by the pandemic, re-deployment, working hours hindering sufficient recovery between shifts and exposure to critical incidents. The study focused on a cohort of experienced RNs who graduated from their nursing programs between 2002 and 2006, meaning that 15–19 years had passed since their graduation. It remains uncertain whether these nurses benefited from their years of experience in dealing with this novel and uncertain situation or if less experienced nurses encountered even more severe challenges. However, it is noteworthy that the prevalence of RNs exhibiting symptoms indicative of possible posttraumatic stress disorder was 3.9 %, which can be compared to the cross-national lifetime prevalence of posttraumatic stress disorder, which stands at 3.9 % in the total sample of World Mental Health Surveys and 5.6 % among the trauma exposed (Koenen et al., 2017). Koenen et al. (2017) considered this 3.9 % a high incidence of lifetime trauma, particularly given that half of all global cases were persistent. In our study, we observed the same prevalence for current trauma, which indicates how posttraumatic stress disorder symptoms affected the RNs during extremely challenging events over a period of 1 year and 9 months of health care work. The overall prevalence of severe posttraumatic stress disorder symptoms among healthcare personnel working during the COVID-19 pandemic, as estimated by a meta-analysis, was 14 % (Andhavarapu et al., 2022), a figure significantly higher than our own estimate. However, in this meta-analysis, there was a notable discrepancy in the outcomes reported between studies utilising the Impact of event scale - revised scale and studies utilising the PCL-5 scale. Specifically, studies employing the PCL-5 scale found a lower prevalence of posttraumatic stress disorder symptoms compared to those using the Impact of event scale - revised scale. It was recommended that future studies regarding the prevalence of posttraumatic stress disorder symptoms among healthcare workers adopt the PCL-5 scale to ensure a more uniform and consistent assessment that enables meaningful comparisons in prevalence studies.

The current COVID-19 pandemic, although it falls outside the conventional definition of trauma that triggers symptoms of posttraumatic stress disorder, has been proposed as a potential traumatic stressor due to various factors, such as the uncertainty surrounding its duration, the fear of illness and death for oneself or loved ones, media coverage, and other issues (Bridgland et al., 2021). Thus, there is a growing body of research indicating the manifestation of traumatic stress symptoms in response to the COVID-19 pandemic as a prevailing global stressor. Even though healthcare professionals regularly encounter death and injury in their line of work, the pandemic brings along added uncertainty and increased patient volume. Throughout the COVID-19 pandemic, nurses have experienced trauma from multiple sources simultaneously, and it is known that nurses are at risk of developing acute stress disorder or post-traumatic stress disorder in response to occupational trauma (Li et al., 2021).

Current frameworks mainly focus on past life-threatening experiences, but growing evidence suggests that individuals can develop traumatic stress reactions from future encounters, indirect experiences, or events not fitting traditional criteria (Bridgland et al., 2021). This implies that work during the COVID-19 pandemic may elicit symptoms of post-traumatic stress disorder, as demonstrated in this study. In line with Koenen et al. (2017) recommendation to enhance access to effective treatment, and considering the prevalence of critical incidents in nursing work and their impact on posttraumatic stress disorder symptom levels, it would be sensible to offer access to treatment within healthcare organisations as part of occupational health and safety initiatives.

To understand why redeployment in the present study showed a significant association with higher levels of posttraumatic stress disorder symptoms in the re-deployed group, Kissel et al. (2023) offer valuable insights based on a sample of nurses with diverse levels of nursing experience. According to their study, a substantial 60 % of redeployed nurses disagreed with the notion of voluntary redeployment. The problem with redeployment seems to be rooted in insufficient preparation, as only 45 % of the nurses agreed that they had received sufficient education and training during the rapid redeployment process (Kissel et al., 2023). Furthermore, Vera San Juan et al. (2022) highlighted lack of support as a significant problem in relation to redeployment when examining the experiences of healthcare workers in intensive care units. These healthcare workers reported substantial anxiety and stress, particularly when faced with inadequate support or insufficient personal protective equipment. The systematic review also emphasised that night shifts posed challenges and last-minute changes in work schedules (Vera San Juan et al., 2022). Redeployment may also be used as a strategy when staff shortages are present even after the peak of the COVID-19 pandemic. In order to learn from health care work during a pandemic, and in order to handle things better in the future and avoid the negative effects of redeployment, Couper et al. (2022) showed that redeployment without adequate or any training was significantly associated with a higher likelihood of experiencing symptoms of posttraumatic stress disorder. The implications of this could be to develop evidence-based ways to meticulously plan their preparation to mitigate the adverse effects of redeployment on employee posttraumatic stress disorder symptoms.

Another finding was that participants who reported working hours hindering sufficient recovery between shifts also had higher levels of trauma symptoms. Previous researchers have shown that incomplete recovery after repeated exposure to stressful situations predicts burnout symptoms (Geurts and Sonnentag, 2006; Shoman et al., 2021), and some have indicated that specific schedule factors (e.g., quick returns to work) can inhibit efficient recovery for health care personnel (Dahlgren et al., 2016; Djupedal et al., 2022). We found that trauma symptoms may play a potential role in the recovery process. This is supported by the observed association between retrospective reports of the degree of exposure to working hours hindering sufficient recovery between shifts and the levels of posttraumatic stress disorder symptoms. For instance, a nurse who experienced recurrent intrusive memories related to critical incidents at work might engage in repeated unsuccessful attempts of thought suppression (a form of avoidance behaviour) which, in turn, might lead to impaired executive functioning and less effective recovery behaviour; i.e., it becomes harder to focus on other areas of life, such as hobbies and personal relationships when constantly reminded of traumatic events at work (Buckley et al., 2000; Qureshi et al., 2011; Shipherd and Beck., 2005). One suggestion for future studies could, therefore, be to conduct in-depth qualitative interviews with RNs to investigate their experiences of exposure to critical incidents and trauma symptoms and determine if and how these symptoms affect the recovery process after a work shift.

In a study by Adriaenssens et al. (2012), more than 80 % of emergency nurses were regularly confronted with work-related critical incidents, with the most distressing event being the death or severe injury of a child or adolescent. This can be compared to our results, where 85% of nurses working across the entire health care system during a pandemic were exposed to critical incidents. The general nurse population may be less exposed to critical incidents than emergency nurses, but, in this study, during a pandemic, we found that critical incidents were prevalent across health care settings. Buhlmann et al. (2021) showed in a systematic review and qualitative synthesis that intensive care nurses, nurses in emergency departments and midwives were exposed to unpredictable, sudden, and traumatic events at work that were destructive and had a professional impact.

In this study, we showed that it was common for the RNs to be exposed to various critical incidents. Overall, exposure to critical incidents was consistently found to be associated with higher levels of posttraumatic stress disorder symptoms. These associations often reflected moderate to high effect sizes for symptoms of intrusive memories or flashbacks and avoidance of trauma-related stimuli. Researchers have shown that both intrusive memories and avoidance behaviour are closely connected with other impairing symptoms (Bryant et al., 2017; Gil and Weinberg, 2015; Harvey and Bryant, 1998; Holmes et al., 2017; Kumpula et al., 2011; North et al., 1999; O'Donnell et al., 2007; Pineles et al., 2011). It is, therefore, interesting that we found in this study that the dose-response effects of critical incidents were specifically related to these two specific symptom clusters. This finding aligns with Buhlmann et al. (2021) discovery that nurses and midwives often employ avoidance strategies to alleviate ongoing distress following critical incidents. Instead of directly addressing the underlying issues, they tend to either avoid certain care practices or attempt to hide problems. The finding of avoidance behaviour, one of the posttraumatic stress disorder symptoms clusters, raises new important questions. For instance, it could be possible that some aspects of the general stress and burnout symptoms commonly experienced by RNs (Rudman et al., 2020; Rudman et al., 2014) are, in fact, driven by intrusive memories or excessive avoidance. One practical recommendation for clinics could, therefore, be to innovate low-threshold assessment procedures that can easily be applied in daily practice and investigate if intrusive memories or avoidance drives the chronification of various mental health problems for these professionals. Another innovation could be to develop brief interventions that can be swiftly provided to those RNs recently exposed to critical incidents. Previous studies have shown that condensed psychological interventions provided in the early aftermath of traumatic events can be effective in reducing both avoidance and intrusive memories (Bragesjö et al., 2023; Iyadurai et al., 2023). An important step could be to adapt these interventions to a clinical context that can be provided to RNs who have experienced a critical incident during the shift. Future studies should also investigate if reductions in these two symptom clusters (intrusive memories and avoidant behaviour) could through psychological interventions have an additional positive “knock-on” effect on other mental health problems and even prevent the development of psychiatric disorders and symptoms of burnout.

### 4.1 Limitations

One limitation of this study is that all data used are self-reported, which can introduce various types of bias. An inherent limitation associated with self-reported data is the possibility that participants may have misunderstood the measurement or provided answers they believed to be desirable, even when the survey is anonymous (Rosenman et al., 2011). However, a strength of self-reported data is that they offer an effective way to collect data from diverse samples. In cases where the phenomenon under investigation, such as experiencing a critical incident, cannot be directly observed and would be unethical to recreate, self-reported data are both appropriate and valuable. The use of self-reported data is essential in behavioural and medical research and is a robust predictor of mortality (Ganna and Ingelsson, 2015).

While nurses demonstrated their ability to recall and report how much their work was affected by the pandemic and recount their exposure to re-deployment and hindrances to recovery between shifts during our pretesting of the instrument, this was not without its limitations. Our objective was to obtain a comprehensive understanding of the work situations faced by experienced nurses throughout the entire pandemic, while simultaneously assessing the consequences or after-effects of working under the pressures introduced by the pandemic. This approach involved a conscious compromise to balance posing numerous questions and avoiding overburdening the respondents. Acknowledging that this limitation may introduce a systematic error if individuals inaccurately recall past events or experiences is crucial. Over time, the accuracy of recalling specific details about past exposures may diminish. Recognising the potential for recall bias, we implemented strategies such as testing the time gap between exposure and assessment, providing multiple options for the same period, and allowing respondents to include remarks in an open-ended question.

In this study, the PCL-5 instrument was completed by nurses without the presence of a person to provide instructions or assist in defining one or more specific traumatic events to keep in mind while filling it in. From the pilot study, we were confident that this instructional approach was effective, and the collected data yielded several indicators that the instructions were clear and followed. Nevertheless, it is essential to acknowledge the potential influence of participation bias (Elston, 2021). It is plausible that individuals experiencing the most severe symptoms might have faced challenges in responding to the survey, potentially resulting in an underestimation of posttraumatic stress disorder symptom prevalence. Conversely, it is also conceivable that RNs with more pronounced symptoms felt a heightened motivation to participate and respond. Due to the longitudinal nature of the LANE study, with many waves of earlier data collections, we were able to determine that the general health of responders unavailable for participation in this wave of data collection (measured by a single item: self-rated health) did not differ from responders, thus reducing to the likelihood of significant selection bias.

## 5 Conclusion

We highlighted the significant impact that working conditions, such as redeployment and exposure to critical incidents during the COVID-19 pandemic, have on the mental health of RNs. The higher levels of posttraumatic stress disorder symptoms among the RNs underscore the need for addressing and mitigating the negative effects of such working conditions. This study contributes to our understanding of the challenges nurses face during the pandemic and emphasises the importance of providing support and interventions to prevent and handle critical incidents at work, promoting their well-being. We also highlighted the importance of thorough planning for workforce readiness in anticipation of future pandemics and other challenging healthcare scenarios, such as staff shortages.

## Other information

No conflict of interest.

## Funding

AFA Insurance grant (Grant nr: 200311) funded this study.

## CRediT authorship contribution statement

**Sara Melander:** Writing – original draft, Formal analysis, Data curation, Conceptualization. **Oili Dahl:** Writing – review & editing, Investigation, Funding acquisition, Conceptualization. **Ann-Charlotte Falk:** Writing – review & editing, Investigation, Funding acquisition, Conceptualization. **Veronica Lindström:** Writing – review & editing, Investigation, Funding acquisition, Conceptualization. **Erik Andersson:** Writing – review & editing, Methodology, Conceptualization. **Petter Gustavsson:** Writing – review & editing, Methodology, Investigation, Formal analysis, Conceptualization. **Ann Rudman:** Writing – review & editing, Supervision, Methodology, Investigation, Funding acquisition, Formal analysis, Data curation, Conceptualization.

## Declaration of competing interest

The authors declare that they have no known competing financial interests or personal relationships that could have appeared to influence the work reported in this paper.

Sara Melander, Oili Dahl, Ann-Charlotte Falk, Veronica Lindström, Erik Andersson, Petter Gustavsson, Ann Rudman.

## Appendix Supplementary materials

Image, application 1
