# Supplementary material for: Critical incidents and post-traumatic stress symptoms among experienced registered nurses during the COVID-19 pandemic: A cross-sectional study
Source: Int J Nurs Stud Adv. 2024 Mar 27;6:100194. doi: 10.1016/j.ijnsa.2024.100194 (PMC11080354; doi:10.1016/j.ijnsa.2024.100194)
Supplement: Supplementary file 1 [file mmc1.docx]

**Supplementary materials.**

Participants in the registered nurses’ cohorts,

number of participants=4316

Excluded number of participants=358,

*Reason:* Declined participation at earlier collections, deceased or moved abroad.

Eligible for participation in follow-up in 2021, i.e., 15-19 years post-graduation,

number of participants=3958

Number of participants consenting to participate=2237

Number of participants not responding=1721

Registered nurses defined as working in health care during the COVID-19 pandemic,

number of participants =1923

Excluded, number of participants=314

*Reason*: Did not work during the COVID-19 pandemic.

Figure S1. Flow chart of recruitment and participation for the cohort study data collection in 2021.

Table S1. Questionnaire items related to COVID-19 work were all repeated for 3-month retrospective periods

(March-May 2020, June-Aug 2020, Sep-Nov 2020, Dec 2020-Feb 2021, March-May 2021, June-Aug 2021).

| **The three main questions related to COVID-19** **work** | **Response alternatives** |
| --- | --- |
|  |  |
| 1) Has your work been affected by the pandemic (during the following time periods)? | Yes, to a great extent, Yes, to some extent, or No, not at all. |
| 2) Have you worked in a different care unit over the following time-periods during the pandemic? | Yes, ordered to, Yes, voluntarily and No. |
| 3) Have your working schedules enabled enough recovery (sleep and opportunity for unwinding) during these periods of the pandemic? | Yes, definitely enough, Yes, generally much enough, No, somewhat insufficient, No, insufficient, No, far from enough |

Table S2. Demographic characteristics of the participants (the sample ranged between 1871-1923 due to

internal dropout).

|  |  |  | Post-traumatic Stress Disorder | |  |  |
| --- | --- | --- | --- | --- | --- | --- |
| **Demographic characteristic**  **of the participants** | n | *%* | Mean | SD | *t/f** | *p* |
| **Gender** |  |  |  |  | 0.589 | 0.556 |
| Female | 1707 | 89.3 | 5.71 | 9.87 |  |  |
| Male | 205 | 10.7 | 5.29 | 8.54 |  |  |
| **Age** |  |  |  |  | 0.465 | 0.628 |
| *<*39 | 217 | 11.3 | 6.03 | 10.70 |  |  |
| 40-49 | 1011 | 52.6 | 5.81 | 10.32 |  |  |
| *>*50 | 694 | 36.1 | 5.42 | 8.62 |  |  |
| **Specialist nurse education** |  |  |  |  |  |  |
| Yes | 1087 | 56.5 | 5.84 | 10.36 | 0.730 | 0.466 |
| No | 836 | 43.5 | 5.51 | 9.00 |  |  |
| **Workplace context** |  |  |  |  | 5.001 | *<*0.001 |
| Nursing ward | 284 | 15.2 | 6.56 | 9.69 |  |  |
| Reception | 502 | 26.8 | 4.55** | 8.65 |  |  |
| Primary health care service | 268 | 14.3 | 3.63** | 7.78 |  |  |
| Nursing home | 346 | 18.5 | 6.37 | 10.16 |  |  |
| Ambulance service | 104 | 5.6 | 5.76 | 8.39 |  |  |
| Operating theatre/intensive care | 267 | 14.3 | 7.93 | 12.18 |  |  |
| Maternity ward | 37 | 2.0 | 5.78 | 8.16 |  |  |
| Research/education | 26 | 1.4 | 9.09 | 16.65 |  |  |
| Other workplace settings | 37 | 2.0 | 7.69 | 13.60 |  |  |
| **Position** |  |  |  |  | 2.483 | 0.059 |
| Registered nurse | 633 | 33.8 | 5.45 | 8.55 |  |  |
| Midwives | 102 | 5.4 | 3.42 | 5.81 |  |  |
| Specialist nurses | 890 | 47.5 | 6.11 | 10.72 |  |  |
| Alternative work in health care | 248 | 13.2 | 5.45 | 10.03 |  |  |
| **Employer** |  |  |  |  | 1.623 | 0.166 |
| Region | 1167 | 63.3 | 5.94 | 9.96 |  |  |
| Municipality | 335 | 18.2 | 6.20 | 10.23 |  |  |
| Nurse agency | 311 | 16.9 | 4.57 | 9.28 |  |  |
| University | 21 | 1.1 | 7.89 | 8.62 |  |  |
| Pharmaceuticals | 9 | 0.5 | 4.44 | 5.39 |  |  |
| **Types of employment** |  |  |  |  | 1.093 | 0.351 |
| Permanent | 1736 | 92.8 | 5.75 | 9.88 |  |  |
| Hourly | 72 | 3.8 | 4.31 | 6.42 |  |  |
| Temporary | 39 | 2.1 | 6.30 | 11.18 |  |  |
| Other types | 24 | 1.3 | 8.33 | 13.69 |  |  |

*All tests are ANOVA (one-way analysis of variance) except the comparison of gender and specialist education (*t*-Test). ****Post-hoc tests: Workplace context, reception of care and primary health care service had a significantly lower mean of post-traumatic stress disorder symptoms in comparison to nursing ward, nursing home, operation/intensive care, research/education and other workplace settings.

Table S3. Levels of exposure to critical incidents and differences in the levels of post-traumatic stress symptoms (post-traumatic stress disorder symptoms total scale scores). Item labels are presented in the manuscript (Table 3).

| **post-traumatic stress disorder sum** | **No exposure^0^** | **One time^1^** | **A few times^2^** | **Many times^3^** |  |  |  | **Effect size** |  |
| --- | --- | --- | --- | --- | --- | --- | --- | --- | --- |
| **Item** | **Mean (SD)** | **Mean (SD)** | **Mean (SD)** | **Mean (SD)** | ***F*** | ***p*** | **0 vs 1** | **0 vs 2** | **= vs 3** |
| 1. | 4.64 (8.81) | 9.45 (11.73) | 8.70 (10.22) | 11.56 (15.33) | 29.653 | *<*.001 | 0.529 | 0.452 | 0.743 |
| 2. | 5.01 (9.03) | 8.62 (12.10) | 7.40 (10.67) | 10.34 (14.17) | 14.371 | *<*.001 | 0.391 | 0.260 | 0.567 |
| 3. | 4.56 (8.79) | 7.47 (11.47) | 7.01 (9.51) | 10.59 (13.46) | 22.736 | *<*.001 | 0.529 | 0.274 | 0.640 |
| 4. | 3.88 (8.01) | 6.50 (9.58) | 8.91 (11.46) | 11.31 (14.77) | 39.967 | *<*.001 | 0.313 | 0.557 | 0.849 |
| 5. | 3.37 (7.12) | 6.82 (9.45) | 7.68 (9.67) | 15.11 (16.38) | 90.744 | *<*.001 | 0.453 | 0.554 | 1.311 |
| 6. | 4.66 (8.58) | 7.89 (11.08) | 8.35 (11.39) | 13.57 (16.19) | 31.417 | *<*.001 | 0.365 | 0.412 | 0.973 |
| 7. | 3.81 (7.78) | 8.08 (9.88) | 11.60 (12.67) | 18.31 (17.38) | 88.467 | *<*.001 | 0.525 | 0.904 | 1.736 |
| 8. | 2.85 (6.36) | 5.99 (8.05) | 8.36 (11.21) | 15.06 (15.84) | 98.625 | *<*.001 | 0.465 | 0.667 | 1.473 |
| 9. | 2.85 (6.49) | 5.86 (8.67) | 7.60 (10.35) | 14.71 (15.32) | 95.129 | *<*.001 | 0.430 | 0.589 | 1.396 |
| 10. | 3.90 (7.58) | 9.06 (11.83) | 10.97 (12.27) | 18.54 (19.11) | 79.192 | *<*.001 | 0.626 | 0.837 | 1.796 |
| 11. | 3.86 (7.49) | 8.91 (11.13) | 11.29 (13.05) | 19.53 (18.38) | 89.025 | *<*.001 | 0.625 | 0.881 | 1.940 |
| 12. | 3.40 (6.87) | 7.93 (11.36) | 9.06 (11.30) | 15.36 (16.11) | 84.447 | *<*.001 | 0.588 | 0.699 | 1.487 |
| 13. | 2.98 (6.67) | 5.30 (8.83) | 6.36 (9.85) | 10.23 (13.07) | 50.762 | *<*.001 | 0.327 | 0.415 | 0.777 |
| 14. | 4.10 (8.30) | 11.13 (15.00) | 10.32 (10.27) | 12.75 (14.33) | 28.113 | *<*.001 | 0.816 | 0.738 | 0.998 |

Table S4. Levels of exposure to critical incidents and differences in the levels of intrusive memories (post-traumatic stress disorder symptoms subscale). Item labels are presented in the manuscript (Table 3).

| **Intrusive memories** | **No exposure^0^** | **One time^1^** | **A few times^2^** | **Many times^3^** |  |  |  | Effect size |  |
| --- | --- | --- | --- | --- | --- | --- | --- | --- | --- |
| **Item (critical incident)** | **Mean (SD)** | **Mean (SD)** | **Mean (SD)** | **Mean (SD)** | ***F*** | ***p*** | **0 vs 1** | **0 vs 2** | **= vs 3** |
| 1. | 0.20 (0.44) | 0.47 (0.61) | 0.45 (0.58) | 0.61 (0.87) | 38.712 | *<*.001 | 0.585 | 0.552 | 0.866 |
| 2. | 0.21 (0.45) | 0.44 (0.63) | 0.38 (0.59) | 0.58 (0.83) | 24.085 | *<*.001 | 0.471 | 0.360 | 0.755 |
| 3. | 0.19 (0.44) | 0.34 (0.58) | 0.34 (0.52) | 0.57 (0.57) | 30.623 | *<*.001 | 0.323 | 0.328 | 0.766 |
| 4. | 0.16 (0.40) | 0.31 (0.48) | 0.44 (0.63) | 0.59 (0.83) | 46.8563 | *<*.001 | 0.352 | 0.602 | 0.948 |
| 5. | 0.12 (0.36) | 0.33 (0.48) | 0.38 (0.50) | 0.79 (0.88) | 111.072 | *<*.001 | 0.547 | 0.656 | 1.433 |
| 6. | 0.19 (0.43) | 0.42 (0.54) | 0.45 (0.23) | 0.78 (0.93) | 54.411 | *<*.001 | 0.514 | 0.575 | 1.257 |
| 7. | 0.16 (0.41) | 0.38 (0.49) | 0.57 (0.65) | 0.98 (0.97) | 92.916 | *<*.001 | 0.504 | 0.902 | 1.837 |
| 8. | 0.10 (0.31) | 0.27 (0.42) | 0.42 (0.59) | 0.78 (0.85) | 111.674 | *<*.001 | 0.483 | 0.737 | 1.569 |
| 9. | 0.10 (0.32) | 0.26 (0.44) | 0.38 (0.56) | 0.75 (0.80) | 106.410 | *<*.001 | 0.454 | 0.666 | 1.476 |
| 10. | 0.17 (0.39) | 0.46 (0.65) | 0.51 (0.59) | 0.97 (1.11) | 77.616 | *<*.001 | 0.659 | 0.786 | 1.843 |
| 11. | 0.16 (0.38) | 0.42 (0.60) | 0.57 (0.68) | 1.03 (1.02) | 93.298 | *<*.001 | 0.598 | 0.924 | 2.035 |
| 12. | 0.14 (0.37) | 0.39 (0.62) | 0.44 (0.58) | 0.70 (0.85) | 71.972 | *<*.001 | 0.599 | 0.685 | 1.289 |
| 13. | 0.12 (0.33) | 0.23 (0.45) | 0.29 (0.51) | 0.50 (0.72) | 48.033 | *<*.001 | 0.306 | 0.400 | 0.752 |
| 14. | 0.17 (0.43) | 0.56 (0.75) | 0.55 (0.56) | 0.74 (0.83) | 40.104 | *<*.001 | 0.879 | 0.885 | 1.253 |

Table S5. Levels of exposure to critical incidents and differences in the levels of avoidance (Post-traumatic Stress Disorder symptoms subscale). Item labels are presented in the manuscript (Table 3).

| **Avoidance behavior** | **No exposure^0^** | **One time^1^** | **A few times^2^** | **Many times^3^** |  |  |  | Effect size |  |
| --- | --- | --- | --- | --- | --- | --- | --- | --- | --- |
| **Item (critical incident)** | **Mean (SD)** | **Mean (SD)** | **Mean (SD)** | **Mean (SD)** | ***F*** | ***p*** | **0 vs 1** | **0 vs 2** | **= vs 3** |
| 1. | 0.17 (0.52) | 0.43 (0.76) | 0.37 (0.67) | 0.66 (1.10) | 27.159 | *<*.001 | 0.470 | 0.360 | 0.848 |
| 2. | 0.19 (0.55) | 0.43 (0.77) | 0.32 (0.70) | 0.50 (0.90) | 12.794 | *<*.001 | 0.421 | 0.216 | .0532 |
| 3. | 0.18 (0.54) | 0.31 (0.66) | 0.27 (0.60) | 0.51 (0.88) | 15.166 | *<*.001 | 0.224 | 0.166 | 0.553 |
| 4. | 0.13 (0.46) | 0.30 (0.62) | 0.39 (0.76) | 0.53 (0.97) | 28.283 | *<*.001 | 0.327 | 0.466 | 0.756 |
| 5. | 0.11 (0.41) | 0.26 (0.56) | 0.33 (0.64) | 0.81 (1.09) | 79.376 | *<*.001 | 0.356 | 0.472 | 1.271 |
| 6. | 0.18 (0.52) | 0.34 (0.65) | 0.37 (0.77) | 0.69 (1.04) | 25.039 | *<*.001 | 0.303 | 0.345 | 0.916 |
| 7. | 0.13 (0.44) | 0.34 (0.65) | 0.55 (0.83) | 1.11 (1.25) | 81.919 | *<*.001 | 0.442 | 0.821 | 1.954 |
| 8. | 0.09 (0.36) | 0.23 (0.53) | 0.35 (0.70) | 0.80 (1.07) | 75.739 | *<*.001 | 0.345 | 0.535 | 1.358 |
| 9. | 0.08 (0.36) | 0.21 (0.21) | 0.34 (0.67) | 0.73 (1.05) | 70.298 | *<*.001 | 0.325 | 0.512 | 1.218 |
| 10. | 0.14 (0.46) | 0.42 (0.79) | 0.49 (0.76) | 1.01 (1.28) | 59.719 | *<*.001 | 0.554 | 0.683 | 1.718 |
| 11. | 0.14 (0.46) | 0.39 (0.74) | 0.52 (0.82) | 1.02 (1.20) | 61.501 | *<*.001 | 0.494 | 0.716 | 1.746 |
| 12. | 0.11 (0.42) | 0.38 (0.78) | 0.40 (0.71) | 0.66 (0.99) | 50.198 | *<*.001 | 0.539 | 0.572 | 1.107 |
| 13. | 0.09 (0.39) | 0.21 (0.56) | 0.25 (0.58) | 0.49 (0.86) | 37.688 | *<*.001 | 0.271 | 0.316 | 0.667 |
| 14. | 0.15 (0.51) | 0.52 (0.86) | 0.56 (0.79) | 0.46 (0.79) | 18.776 | *<*.001 | 0.707 | 0.774 | 0.596 |

Table S6. Levels of exposure to critical incidents and differences in the levels of negative emotions and mood (Post-traumatic Stress Disorder symptoms subscale). Item labels are presented in the manuscript (Table 3).

| **Negative emotions and mood** | **No exposure^0^** | **One time^1^** | **A few times^2^** | **Many times^3^** |  |  |  | Effect size |  |
| --- | --- | --- | --- | --- | --- | --- | --- | --- | --- |
| **Item (Critical incident)** | **Mean (SD)** | **Mean (SD)** | **Mean (SD)** | **Mean (SD)** | ***F*** | ***p*** | **0 vs 1** | **0 vs 2** | **= vs 3** |
|  | 0.22(0.49) | 0.44(0.63) | 0.40(0.55) | 0.56(0.86) | 21.034 | *<*.001 | 0.436 | 0.353 | 0.658 |
|  | 0.24(0.51) | 0.39(0.66) | 0.32(0.55) | 0.48(0.72) | 8.411 | *<*.001 | 0.290 | 0.156 | 0.456 |
|  | 0.22 (0.49) | 0.36(0.61) | 0.32(0.52) | 0.48(0.71) | 13.561 | *<*.001 | 0.271 | 0.197 | 0.487 |
|  | 0.19(0.45) | 0.31(0.52) | 0.41(0.62) | 0.53(0.81) | 26.541 | *<*.001 | 0.255 | 0.442 | 0.707 |
|  | 0.16(0.39) | 0.31(0.54) | 0.36(0.55) | 0.71(0.90) | 63.905 | *<*.001 | 0.347 | 0.465 | 1.120 |
|  | 0.23(0.48) | 0.34(0.61) | 0.36(0.59) | 0.62(0.88) | 18.351 | *<*.001 | 0.240 | 0.280 | 0.774 |
|  | 0.18(0.43) | 0.37(0.54) | 0.54(0.70) | 0.84(1.00) | 57.917 | *<*.001 | 0.415 | 0.745 | 1.388 |
|  | 0.14(0.37) | 0.26(0.41) | 0.40(0.62) | 0.71(0.88) | 69.465 | *<*.001 | 0.319 | 0.557 | 1.204 |
|  | 0.14(0.37) | 0.25(0.47) | 0.34(0.55) | 0.73(0.87) | 71.291 | *<*.001 | 0.285 | 0.452 | 1.208 |
|  | 0.18(0.42) | 0.41(0.62) | 0.54(0.70) | 0.92(1.02) | 62.585 | *<*.001 | 0.514 | 0.747 | 1.614 |
|  | 0.18(0.42) | 0.42(57) | 0.52(0.73) | 0.93(1.06) | 62.871 | *<*.001 | 0.534 | 0.720 | 1.621 |
|  | 0.15(0.38) | 0.36(0.59) | 0.43(0.61) | 0.81(0.92) | 76.159 | *<*.001 | 0.494 | 0.622 | 1.446 |
|  | 0.14(0.38) | 0.24(0.43) | 0.31(0.54) | 0.48(0.71) | 38.089 | *<*.001 | 0.253 | 0.369 | 0.665 |
|  | 0.19(0.46) | 0.51(0.81) | 0.47(0.53) | 0.57(0.74) | 17.866 | *<*.001 | 0.649 | 0.592 | 0.787 |

Table S7. Levels of exposure to critical incidents and differences in the levels of physiological activation (Post-traumatic Stress Disorder symptoms subscale). Item labels are presented in the manuscript (Table 3).

| **Physiological activation** | **No exposure^0^** | **One time^1^** | **A few times^2^** | **Many times^3^** |  |  |  | Effect size |  |
| --- | --- | --- | --- | --- | --- | --- | --- | --- | --- |
| **Item (Critical incident)** | **Mean (SD)** | **Mean (SD)** | **Mean (SD)** | **Mean (SD)** | ***F*** | ***p*** | **0 vs 1** | **0 vs 2** | **= vs 3** |
| 1. | 0.28 (0.52) | 0.54 (0.67) | 0.48 (0.48) | 0.53 (0.73) | 17.743 | *<*.001 | 0.479 | 0.360 | 0.460 |
| 2. | 0.30 (0.54) | 0.47 (0.61) | 0.42 (0.61) | 0.50 (0.68) | 7.467 | *<*.001 | 0.303 | 0.215 | 0.356 |
| 3. | 0.27 (0.52) | 0.43 (0.64) | 0.42 (0.57) | 0.55 (0.68) | 16.909 | *<*.001 | 0.299 | 0.273 | 0.516 |
| 4. | 0.24 (0.47) | 0.36 (0.58) | 0.51 (0.66) | 0.59 (0.75) | 29.347 | *<*.001 | 0.243 | 0.498 | 0.683 |
| 5. | 0.23 (0.45) | 0.40 (0.54) | 0.42 (0.57) | 0.74 (0.87) | 50.616 | *<*.001 | 0.362 | 0.399 | 0.965 |
| 6. | 0.29 (0.51) | 0.45 (0.66) | 0.45 (0.63) | 0.64 (0.77) | 16.369 | *<*.001 | 0.298 | 0.311 | 0.660 |
| 7. | 0.23 (0.46) | 0.48 (0.62) | 0.63 (0.70) | 0.91 (0.90) | 63.674 | *<*.001 | 0.495 | 0.787 | 1.395 |
| 8. | 0.19 (0.40) | 0.39 (0.55) | 0.46 (0.63) | 0.76 (0.83) | 63.828 | *<*.001 | 0.450 | 0.545 | 1.344 |
| 9. | 0.19 (0.41) | 0.38 (0.57) | 0.43 (0.58) | 0.73 (0.82) | 59.681 | *<*.001 | 0.427 | 0.489 | 1.080 |
| 10. | 0.24 (0.46) | 0.50 (0.65) | 0.60 (0.71) | 0.86 (0.96) | 52.217 | *<*.001 | 0.526 | 0.704 | 1.268 |
| 11. | 0.24 (0.46) | 0.51 (0.63) | 0.62 (0.72) | 0.97 (0.95) | 65.056 | *<*.001 | 0.560 | 0.765 | 1.513 |
| 12. | 0.22 (0.43) | 0.44 (0.60) | 0.50 (0.65) | 0.80 (0.88) | 57.077 | *<*.001 | 0.460 | 0.559 | 1.181 |
| 13. | 0.19 (0.41) | 0.33 (0.51) | 0.37 (0.56) | 0.56 (0.73) | 38.074 | *<*.001 | 0.319 | 0.373 | 0.668 |
| 14. | 0.26 (0.49) | 0.63 (0.83) | 0.53 (0.57) | 0.68 (0.82) | 19.060 | *<*.001 | 0.735 | 0.554 | 0.816 |
